# Engagement in the National Diabetes Prevention Program Among Hispanic Men

**DOI:** 10.1001/jamanetworkopen.2025.15046

**Published:** 2025-06-18

**Authors:** Christopher J. Gonzalez, Clarence N. Perez-Mejia, Noelia Hernandez, Hana Flaxman, Cara Stephenson-Hunter, Eric Gil, Elaine De Leon, Taynara Formagini, Earle C. Chambers, Martin F. Shapiro, Jeffrey S. Gonzalez

**Affiliations:** 1 Division of General Internal Medicine, Weill Cornell Medical College, New York, New York; 2 MD Program, Weill Cornell Medicine, New York, New York; 3 Harold and Muriel Block Institute for Clinical and Translational Research, Albert Einstein College of Medicine, Bronx, New York; 4 Department of Family and Social Medicine, Albert Einstein College of Medicine, Bronx, New York; 5 Department of Medicine, Albert Einstein College of Medicine, Bronx, New York; 6 Department of Epidemiology and Population Health, Albert Einstein College of Medicine, Bronx, New York; 7 Department of Medicine, New York University Grossman School of Medicine, New York; 8 Department of Family Medicine, University of California, San Diego; 9 Ferkauf Graduate School of Psychology, Yeshiva University, Bronx, New York

## Abstract

**Importance:**

Hispanic men experience higher rates of diabetes compared with non-Hispanic White men but remain underrepresented in preventive lifestyle interventions.

**Objective:**

To identify unique reasons for Hispanic men’s low engagement in the National Diabetes Prevention Program (NDPP) compared with those with high engagement to inform strategies for improving participation.

**Design, Setting, and Participants:**

This qualitative study recruited Hispanic men aged 18 years and older and at risk for diabetes per electronic medical records from June 2023 to February 2024 from primary care sites affiliated with hospital-associated Montefiore Health Systems related to an ongoing clinical trial called Power Up. Patients were invited to participate in one-time phone interviews. Interviews were analyzed deductively, guided by behavior change frameworks.

**Exposure:**

Participant’s level of engagement varied with representation balanced between those demonstrating low and high engagement in the NDPP (attendance at <4 and ≥4 sessions, respectively).

**Main Outcomes and Measures:**

Planned study outcomes included NDPP notable factors associated with low engagement.

**Results:**

Of 32 Hispanic men who completed interviews (20 patients [62.5%]aged >50 years), 15 had low engagement in the NDPP, 13 (87%) were not born in the US, 12 (80%) had limited English proficiency, and 8 (53%) did not complete high school. Three major themes and 11 subthemes emerged. Hispanic men with low engagement discussed limited awareness of their prediabetes diagnosis, lifestyle change self-sufficiency, and skepticism about their diabetes risk and the benefits of NDPP. Also mentioned were financial barriers, restricted access to the program, and the perceived negatives of participation outweighing potential benefits.

**Conclusions and Relevance:**

Addressing the unique barriers faced by Hispanic men in engaging with the NDPP is critical to reducing diabetes-related inequities and may require tackling knowledge gaps, financial barriers, and perceptions of program relevance before, during, and after enrollment. Future research should explore how to tailor recruitment strategies and program content to Hispanic men’s specific identities, motivations, and challenges.

## Key Points

**Question:**

What factors in the National Diabetes Prevention Program (NDPP) are associated with in low engagement among Hispanic men?

**Findings:**

The qualitative study of 32 men revealed 3 major themes and 11 subthemes aligning with the major components of behavioral change and theoretical domains framework. Barriers discussed included limited prediabetes diagnosis awareness, lifestyle change self-sufficiency, skepticism around diabetes risk, and the NDPP’s benefits outweighing its risks, financial barriers, and limited program access.

**Meaning:**

These findings suggest improving engagement among Hispanic men in the NDPP may require addressing perceptions about program relevance, financial limitations, and gaps in knowledge around diabetes prevention before, during, and after enrollment.

## Teaser

This qualitative study evaluated reasons for Hispanic men’s low engagement in the National Diabetes Prevention Program (NDPP) compared with those with high engagement.

## Introduction

Hispanic men in the US, a population of 32 million, have a diabetes prevalence 64% higher than non-Hispanic White men (14.3% and 8.7% respectively), along with higher risks of diabetes-related visual impairment, kidney failure, lower extremity amputations, and death, underscoring the critical importance of preventing type 2 diabetes (hereafter referred to as diabetes) in this population.^1,2,3^ The Diabetes Prevention Program (DPP), an evidence-based intensive lifestyle intervention designed to achieve 7% weight reduction, decreased the risk of developing diabetes by 58% in individuals with prediabetes.^4^ Despite attempts for wide dissemination of the DPP through the US Center for Disease Control and Prevention’s National Diabetes Prevention Program (NDPP), Hispanic men compose only 2% of those enrolled nationwide, with only 52.6% maintaining continuous engagement in its 16 core sessions, compared with 70.5% of non-Hispanic White adults enrolled.^5^ Engagement in the NDPP has important implications for its effectiveness: longitudinal participation is linearly associated with the likelihood of achieving the program’s weight loss goal.^5^

Increasing Hispanic men’s engagement in the NDPP could reduce racial disparities by preventing or delaying the onset of diabetes in this group, contributing to a lower prevalence of diabetes, fewer medications, hospitalizations and complications.^5,6,7^ However, little is known about how to better engage Hispanic men in the NDPP.^8,9,10^ Studies exploring barriers to engagement in lifestyle interventions like the NDPP have often overlooked Hispanic men altogether.^11,12^ More recent studies, including the HOMBRE and Animo trials, have substantially included Hispanic men, assessing whether tailoring or adapting weight loss interventions improve their effectiveness.^13,14,15,16^ While these studies provide important evaluative information, they have not employed comprehensive theory-driven approaches that can inform how to better engage Hispanic men in diabetes prevention. Importantly, prior studies often sample Hispanic men with high levels of engagement in the NDPP, potentially missing the valuable perspectives of those with limited engagement in the program.

The study objective was to comprehensively identify factors that potentially differentiate Hispanic men invited to the NDPP who had low levels of engagement from those with substantial engagement. Understanding factors unique to those with low engagement could inform interventions for the purpose of increasing the engagement of Hispanic men in the NDPP.

## Methods

### Setting, Recruitment, and Study Sample

Recruitment for this qualitative descriptive study occurred from June 2023 to February 2024, leveraging standard recruitment protocols for the Power Up study, a separate ongoing clinical trial funded by the National Institutes of Health testing the effectiveness of an NDPP tailored for men.^13^ Power Up used electronic health records (EHR) from primary care sites partnered with the Montefiore Health System and the New York City Department of Health and Mental Hygiene to identify men at risk of diabetes. NDPPs were virtual and offered in English and Spanish. Men screened for Power Up were invited to the current study if they self-identified as Hispanic, were aged 18 years or older, had an HbA_1c_ of 5.7% to 6.4% within the past year, and could be interviewed by phone. Individuals were invited during outreach phone-calls recruiting them to Power Up or after all core sessions occurred.

Participants underwent oral informed consenting by study personnel and received $50 gift cards. Purposeful sampling ensured similar representation of Hispanic men with relatively low vs high engagement, defined, respectively, as attending less than 4 vs 4 or more core sessions. This threshold has been used as a marker of early attrition in prior studies assessing NDPP engagement and was historically used as a marker for reimbursement by insurers.^5,17^ The institutional review board of Weill Cornell Medicine reviewed and approved the study protocol, and Albert Einstein College of Medicine approved the referral process. The study followed the Consolidated Criteria for Reporting Qualitative Research (COREQ) reporting guideline.^18^

Given the recommendations behind exploratory research questions, we aimed for a sample size of 45 participants.^19^ Fifty-five individuals were invited; 32 completed interviews. The remainder declined for lack of interest (12 individuals ) or could not be reached (11 individuals ). Recruitment concluded when no new codes emerged from the interviews.^20^

### Data Collection

A trained qualitative interviewer (C.N.P.; a bilingual Hispanic man) conducted one-on-one interviews (median [IQR] length, 26.4 [18.4-39.1] minutes) via recorded phone calls in English or Spanish. The study used individual interviews to avoid the influence of potential biases in group conversations and provide a space for participants to discuss sensitive information. The interviewer used a semistructured interview guide (eAppendix in Supplement 1) informed by behavior change literature including the theoretical domains framework (TDF) and COM-B, a component of the behavioral change wheel that helps contextualize individual-level change by examining how capabilities, opportunities, and motivation impact behavior.^21^ TDF further stratifies barriers and facilitators of change to 14 domains overlapping with COM-B.^21,22^ The guide explored perceived barriers to and benefits of engaging in a DPP and potentially helpful resources. Given the study focus, probes explored how ethnicity and gender were associated with DPP engagement. Recordings were deidentified and professionally transcribed into English. At the end of the interview, a brief 12-question questionnaire obtained demographic data (variables and measures delineated in Table 1).

**Table 1.** Characteristics of Sample, Engaging Hispanic Men in the Diabetes Prevention Program

Characteristics	Participants, No. (%)		*P* value^a^
	Low engagers (n = 15)	High engagers (n = 17)	
Age, y			
<50	6 (40)	5 (29)	.61
>50	9 (60)	11 (65)	
NA^b^	0	1 (6)	
Nativity			
Not born in US	13 (87)	11 (65)	.23
Hispanic origin^c^			
Colombia	0	2 (12)	.73
Dominican Republic	9 (60)	7 (41)	
Ecuador	1 (7)	0	
Honduras	0	1 (6)	
Mexico	2 (13)	1 (6)	
Puerto Rico	3 (20)	5 (29)	
Venezuela	1 (7)	0	
NA	0	1 (6)	
Language preference^d^			
Spanish	12 (80)	8 (47)	.08
English proficiency^e^			
Not proficient	12 (80)	6 (35)	.02^f^
Education			
Less than high school	8 (53)	2 (12)	.02^f^
Completed high school	4 (27)	5 (29)	
More than high school	3 (20)	10 (59)	
Health literacy^g^			
Good	9 (60)	13 (76)	.45
Comorbidities, No.^h^			
0	2 (13)	4 (24)	.53
1	5 (33)	3 (18)	
≥2	8 (53)	10 (59)	

Abbreviation: NA, not applicable.

^a^ Differences between low and high engagers were analyzed using Fisher exact test and χ^2^ tests.

^b^ NA includes participants that declined to answer or did not apply.

^c^ Hispanic origins were self-reported, and individual participants could identify multiple origins.

^d^ Language preference reflected the participants’ preferred language for the interview.

^e^ English proficiency was measured using the question “How well do you think you speak English?” and categorized into not proficient using responses “not at all,” “not well,” and proficient using “well” or “very well.”

^f^ Indicates *P* < .05.

^g^ Health literacy was measured using the question “How confident are you filling out medical forms by yourself?” and categorized into poor using responses “not at all,” “a little bit,” “somewhat,” and good using “quite a bit” or “extremely.”

^h^ Comorbidities centered on self-reported history of high blood pressure, high cholesterol, any history of cancer, heart problems, liver problems, kidney problems, lung problems, problems with the immune system, or depression and/or anxiety.

### Statistical Analysis

Transcripts were independently coded by 2 researchers using NVivo software version 14.23.4. Codes were reconciled by a third researcher, with discrepancies resolved through discussion with the study team and consolidated into a codebook. Coding primarily used a deductive thematic approach, with new codes categorized into the major constructs of the COM-B and TDF.^22,23^ The study team then compared codes that emerged among Hispanic men with relatively low vs high engagement (see Study Sample section). Similar codes within each major construct of TDF were categorized into belief statements illustrating emerging content. This comparative analysis primarily analyzed codes and belief statements emerging uniquely among Hispanic men with low engagement in the NDPP (henceforth, low engagers) and when available, used contrasting codes and belief statements emerging among those with high engagement (henceforth, high engagers) to provide additional context. Ultimately, 80 codes emerged, presented in the eTable in Supplement 1. Finally, subthemes and themes, largely reflecting the major constructs of TDF and COM-B, respectively, were determined through consensus of the study team. Participants did not review or provide feedback on findings. A Fischer exact test and χ^2^ test were used to analyze *P* values between participants’ characteristics with low and high engagement using Stata software version 16.0. One-sided and 2-sided tests did not qualify for these tests. Significance was set at *P* < .05.

## Results

Among 32 Hispanic men (20 patients [62.5%]aged >50 years) who completed interviews, 13 (87%) were not born in the US, and 15 (47%) were low engagers. Compared with high engagers (Table 1), a greater proportion of low engagers had limited English proficiency (12 participants [80%]), and had not completed high school (8 participants [53%]). Three major themes and 11 subthemes, reflecting the major domains of COM-B and TDF, respectively, delineate the perceived reasons for engagement and their recommendations for the NDPP among low engagers as summarized in Table 2.

**Table 2.** Themes and Subthemes, Engaging Hispanic Men in the Diabetes Prevention Program (DPP)

COM-B domain (theme) and TDF domain (subtheme)^a^^,^^b^	Belief among those with low engagement^c^	Representative quote (participant)	Contrasting belief among those with high engagement^d^
Capabilities			
Knowledge	I have limited awareness of prediabetes and the DPP	“When they called me, they said I had prediabetes. But I think they made a mistake because my doctor didn’t tell me that.” (participant 32)	Did not emerge
	Healthy diets are restrictive diets	“Eating more vegetables than any other things, not eating flour, not eating lots of fat.” (participant 31)	Moderation and balance are healthy
Skills	I can think independently and have useful interpersonal skills	“I watch everything and I come to my own conclusions about what I think.” (participant 28)	Specific skills allow me to eat healthily and exercise
Mental capacity and behavioral regulation	I forget to eat well	“He told me to consume foods like avocado, but I forgot about it because I don’t have symptoms.” (participant 1)	Consistently choosing healthy options can be a cognitive load
Motivations			
Beliefs about capacity	I am self-sufficient	“I am taking care of myself to a degree that I watch what I eat and I don’t feel like I need anything else.” (participant 1)	Did not emerge
Beliefs about consequences	The risks of the DPP outweigh its benefits	“I am always worried there might come a time when I have to get insulin shots.” (participant 13)	Did not emerge
Emotions	I am skeptical of my diagnosis and of doctors	“My doctor didn’t talk to me about prediabetes. I felt uncomfortable, so I got on the internet.” (participant 28)	I fear diabetes
Goals and intentions	I want to better navigate health care	“I want them to inform me how to handle the health care system.” (participant 32)	I want to avoid diabetes
Reinforcement	DPP coaches teach what doctors do not	“A counselor telling you how it is, what’s real about the disease, and that’s what makes me stay.” (participant 22)	Did not emerge
Social professional role/identity	The DPP should consider my Hispanic identity	“You would feel more comfortable if everyone is Hispanic, if everyone speak the same language to understand.” (participant 1)	The DPP should consider my male identity
Opportunities			
Environmental context and resources	Finances impact my access to health care	“I don’t have the money for a physical because I have to pay for the consultation.” (participant 32)	Finances impact my exercise options
	DPP is inaccessible	“It’s not that I don’t want the DPP, they just didn’t call me back.” (participant 22)	Accessing the DPP is challenging but feasible
Social influences	I act when my physicians tell me to act	“The doctor is supposed to tell me what to do about prediabetes, but he hasn’t told me anything, so I live life as if I don’t have anything.” (participant 1)	Did not emerge
	Prediabetes is socially normalized	“Every person I have spoken to says they’re prediabetic and that it’s normal and nothing to be concerned about.” (participant 1)	My friends and/or family have diabetes—I have to prevent it

Abbreviations: COM-B, component of the behavioral change wheel that helps contextualize individual-level change by examining how capabilities, opportunities, and motivation impact behavior; TDF, theoretical domains framework.

^a^ Themes include the COM-B domains, consisting of capabilities, motivations, and opportunities.

^b^ Subthemes include TDF domains and fall within their respective COM-B domain.

^c^ Belief statements were the next categorization to organize low engager codes that were similar within the major constructs of TDF.

^d^ Contrasting beliefs represented high engagers sentiments that were opposite to low engagers belief statements.

### Capacity to Engage in the NDPP and in Related Preventive Behaviors

#### Knowledge

Low engagers expressed having limited awareness that they had been diagnosed with prediabetes, a lack of understanding of its implications, and a limited understanding of what could be done to prevent diabetes. They believed adhering to restrictive diets was necessary to prevent diabetes. In contrast, high engagers exhibited a more nuanced understanding of diabetes prevention, including the importance of adhering to diverse diets that focused on moderation. Notably, participants were interviewed following their engagement in the NDPP.

#### Skills

Low engagers described having useful interpersonal skills, including the ability to discuss health topics with others based on their own lived experiences, and critical thinking skills, including the capacity to independently interpret and make conclusions about health behaviors. In contrast, high engagers described skills related to lifestyle changes, including reading the nutritional labels on food products, monitoring their caloric intake, and executing exercises independently.

#### Mental Capacity

Low engagers described experiencing challenges with remembering to eat healthily, citing that prediabetes was an asymptomatic condition. High engagers highlighted the cognitive load of consistently choosing to eat well, which included controlling urges and cravings.

### Motivations to Engage in the NDPP and in Related Preventive Behaviors

#### Beliefs About Capacity to Engage in Preventive Behaviors

Low engagers expressed feeling self-sufficient regarding managing their own health or enacting lifestyle changes needed to prevent diabetes and described not needing assistance from others to make these changes. Comparable or contrasting beliefs did not emerge among high engagers.

#### Beliefs About the Consequences of Engaging in the NDPP

Unlike high engagers, low engagers felt that the risks of engaging in the NDPP outweighed its potential benefits. They cited financial limitations, including the cost of enrolling in the program and the collateral expenses of attending. Notably, the program offered to participants was free. Low engagers also expressed fears that engaging in a program to prevent diabetes could progressively lead to overtreatment, and ultimately to requiring insulin.

#### Emotions

Low engagers discussed feeling unease about being diagnosed with prediabetes, expressing skepticism about the diagnosis and its implications, as well as about health care practitioners and their motivations. Contrastingly, high engagers discussed how their fear of developing diabetes motivated them to engage in preventive behaviors, including participating in the NDPP.

#### Goals and Intentions

Low engagers described their intentions to better navigate the health care system overall. This included being able to communicate with their health care practitioners and with programs like the NDPP. In contrast, high engagers specifically described their goal of avoiding diabetes.

#### Reinforcing Features of the NDPP

Low engagers felt that one positive attribute of the NDPP is that guides and coaches have time to educate patients about diabetes prevention. They noted that health care practitioners are valuable sources of information, but they often do not have sufficient time, do not focus on prevention, and do not provide specific lifestyle guidance. Comparable or contrasting beliefs did not emerge among high engagers.

#### Identity

Low engagers discussed how their Hispanic identity affected the relevance of the NDPP and their interest in engaging with the program. Specifically, they explained that diabetes was common among Hispanic populations in the US, potentially because of some dietary staples and habits. They also felt that Hispanic adults often have unique learning needs and preferences, including a desire to receive digestible amounts of information at a time, and often avoid the health care system. In contrast, high engagers discussed the relevance of their male identity and its influence on engaging with a NDPP that was tailored to men. Specifically, they noted that camaraderie among individuals in the NDPP was a critical feature encouraging continued engagement in the NDPP, and that men felt more comfortable sharing personal details and being vulnerable among other men.

### Opportunities to Engage in the NDPP and in Related Preventive Behaviors

#### Environmental Context and Resources

Low engagers felt that limited financial resources impacted their access to health care, which in turn limited access to engaging in preventing diabetes. High engagers also discussed economic limitations, but contrastingly, they felt their finances impacted access to engaging in specific preventive behaviors, such as exercise equipment and gym memberships. Low engagers also noted that the NDPP was inaccessible to them, because of either geographic or technological limitations (eg, perceptions that the NDPP is located too remotely or that it requires technological literacy), or because of the complexity of navigating the health care system. In contrast, high engagers also noted that getting in contact with the program could be challenging, but that it was eventually feasible for them.

#### Social Influences

Low engagers felt that diabetes was so common in their social networks that it had essentially been normalized, hindering any urgency to prevent it. However, high engagers felt that diabetes was sufficiently present in their social networks to promote awareness and fear of it, thereby encouraging engagement in diabetes prevention. Low engagers also highlighted the influence of health care practitioners as cues to action, noting that practitioners were responsible for communicating the diagnosis of prediabetes and for conveying what needed to be done to prevent diabetes.

### Patient Suggestions for Facilitating Engagement in Diabetes Prevention Efforts

Regardless of engagement, participants requested educational materials that offered specific guidance for preventing diabetes. They recommended that such materials be distributed digitally or online. They also suggested that the NDPP offer financial incentives to encourage and facilitate engagement in the program. Low engagers specifically requested assistance with health care navigation and recommended that diabetes prevention efforts leverage phone outreach to identify and encourage eligible patients to participate. They also felt that diabetes prevention efforts should be done in group settings (as is done in the NDPP) because social interaction can facilitate engagement and learning.

## Discussion

This qualitative study of eligible Hispanic men invited to the NDPP found several unique factors differentially affecting those that do not substantially attend the program. Uniquely among those with relatively low engagement, participants expressed: (1) persistently limited knowledge of prediabetes and of the NDPP, (2) perceptions of self-sufficiency and skepticism about their diagnosis and of the program, and (3) challenges to continually engaging with lifestyle changes and with the NDPP, including financial and access barriers and discouraging social networks. This comprehensive exploration of factors hindering NDPP engagement among Hispanic men provides important insights about opportunities, including potential intervention targets and strategies, that can be leveraged to better engage them in diabetes prevention (Box).

Box. **Potential Opportunities for Engaging Hispanic Men in the Diabetes Prevention Program (DPP)**

**Provide resources that enable engagement in preventive behaviors**

- Expand availability of DPP and its content through digital formats.
- Supplement the DPP with tailored messages and modeled behaviors that reinforce engagement.
- Incentivize or enable physical activity and healthy diets, such as through gym memberships.

**Address common misconceptions and promote awareness**

- Explain the risks of prediabetes and the importance of lifestyle change to avoid medical treatment.
- Clarify the DPP’s person-centeredness, group format, focus on autonomy, and dedication to prevention.

**Facilitate tailored communication with practitioners and care teams**

- Allocate time to discuss diabetes prevention with at-risk patients.
- Train care teams to have person-centered conversations that encourage engagement in the DPP.
- Implement patient outreach to communicate diabetes risk and preventive goals.

**Engage peers and community health workers in components of care**

- Decide to take part in the DPP.
- Training regarding care navigation, including accessing DPP and other resources.
- Model physical activity and other preventive behaviors.

Awareness about prediabetes and the NDPP is relatively limited among those eligible for the program.^24,25^ However, our findings illustrate that limited awareness persists among Hispanic men despite being informed of their elevated risk and after verbalizing their decision to participate in the NDPP, even in the context of a linguistically concordant protocolized research study. More nuanced context-specific approaches may be needed to effectively communicate diabetes risk and to discuss the NDPP and increase participation in the program among eligible Hispanic men. One promising approach is to include and prepare patients’ usual care teams in the process of recruiting eligible participants. Recruitment in this study was done through phone outreach to eligible individuals identified through the EHR and not by patients’ usual care teams. Echoing prior findings, Hispanic men with low engagement noted that physicians play a vital role in communicating the implications of a diagnosis of prediabetes and offering critical cues to action.^26^ Together, this suggests that patients’ usual care teams may be trusted sources of health information needed in discussions about prediabetes and the NDPP, and necessary to adequately engage Hispanic men in the program.^27^

Hispanic men generally preferred tailored or adapted programs. Low engagers highlighted that tailoring should focus on the unique needs and preferences of Hispanic populations, whereas high engagers discussed the benefits of tailoring the program specifically for men, particularly because it facilitated camaraderie among participants. Cultural and linguistic tailoring is considered vital to adequately engaging historically minoritized communities in behavioral interventions, and prior studies have identified men’s desire for gender-tailored DPPs.^28^ This contrast may have emerged from differences in acculturation or educational attainment between low and high engagers, with the former potentially preferring rigorous tailoring that met their cultural and educational needs. Alternatively, high engagers, having been exposed to more of the NDPP, may have felt it was sufficiently culturally tailored or that gender was a greater influence on the group dynamics critical to the program’s success. Regardless, this contrast highlights the need for the NDPP to be tailored and adapted to meet the heterogeneous needs of individuals at risk for diabetes, at minimum in regards to acculturation, educational attainment, and gender perceptions. While few studies have assessed the effectiveness of tailoring the DPP to these unique needs, those that do have shown promise.^14,16,29^ Importantly, our findings imply tailoring may need to extend beyond the program itself, including recruitment.

Low engagers discussed several barriers hindering engagement, including financial limitations, but noted 2 specific beliefs discouraging them from participating: (1) feeling skepticism about the diagnosis and the program, including that it potentially had more risks than benefits; and (2) feeling that they could successfully enact lifestyle change without the help of a program. Studies show that men are less likely than women to seek or access care overall,^30,31,32^ and relatedly, that they are less likely to engage in lifestyle interventions.^5^ Apprehension to engage with health care may be in part rooted in fear of receiving a diagnosis that would impact daily living and, uniquely for Hispanic men, their perceived roles as head of household.^33,34^ Low engagers specifically expressed fear that the NDPP would lead to additional, and potentially unnecessary, medical management. Future research should explore how to overcome these concerns, including clearly communicating the NDPP’s safety and efficacy, while validating patients’ autonomy and the added value of structured support through the program. By addressing these barriers and focusing on culturally relevant strategies, interventions can better align with the values and preferences of Hispanic men, ultimately improving engagement and outcomes in diabetes prevention efforts.

Several potential facilitators to engaging with the NDPP emerged, offering additional insights about ways to improve engagement among low engagers. Our findings suggest that facilitating health care navigation, highlighting the consequences of developing diabetes, advertising the group format of the program as a resource for staying accountable to their lifestyle change goals, and offering the program in diverse formats are each strategies that could be leveraged to potentially improve enrollment and initial engagement in the program. As means of improving continued engagement in the program, potential strategies that could be leveraged included tailoring the program to meet their specific needs, leveraging tailoring to facilitate camaraderie among NDPP members, and offering resources to overcome financial limitations.

### Limitations

This study has limitations. This theory-informed qualitative study is hypothesis generating; belief statements were not intended to be quantified or prioritized in importance. The participant sample pool was restricted to those engaged in health care and to the Power Up study’s NDPP enrollment procedure. Demographic differences between low and high engagers may have confounded comparative findings. Participant body mass index data were not assessed. High engagers also may have gained knowledge and skills through their NDPP participation, while low engagers likely shared experiences independent of the program. Use of established implementation frameworks (COM-B and TDF) allowed for comprehensively exploring factors influencing engagement, but may have limited the scope of responses acquired.

## Conclusions

In this qualitative study of Hispanic men invited to participate in a NDPP, we revealed potential key differences between those with low and high engagement. Low engagers lacked awareness of prediabetes and preventive measures, felt confident managing lifestyle change independently, and perceived NDPP risks outweighed its benefits. Financial constraints and health care skepticism further hindered engagement. Our findings showed that enhancing engagement among Hispanic men may require addressing knowledge gaps, financial barriers, and perceptions of program relevance, while leveraging culturally tailored approaches that resonate with their identities and motivations.

## Notes

Supplement 1. **eAppendix.** Interview Guide: Engaging Hispanic Men in the Diabetes Prevention Program **eTable. **Codebook: Engaging Hispanic Men in the Diabetes Prevention Program

Supplement 2.
Data Sharing Statement
