# Supplementary material for: Engagement in the National Diabetes Prevention Program Among Hispanic Men
Source: JAMA Netw Open. 2025 Jun 18;8(6):e2515046. doi: 10.1001/jamanetworkopen.2025.15046 (PMC12177656; doi:10.1001/jamanetworkopen.2025.15046)
Supplement: Supplement 1. — eAppendix. Interview Guide: Engaging Hispanic Men in the Diabetes Prevention Program eTable. Codebook: Engaging Hispanic Men in the Diabetes Prevention Program [file jamanetwopen-e2515046-s001.pdf]

Supplemental Online Content

Gonzalez CJ, Perez-Mejia CN, Hernandez N, et al. Engagement in the diabetes prevention program among Hispanic men. *JAMA Netw Open*. 2025;8(6):e2515046. doi:10.1001/jamanetworkopen.2025.15046

eAppendix. Interview Guide: Engaging Hispanic Men in the Diabetes Prevention Program
eTable. Codebook: Engaging Hispanic Men in the Diabetes Prevention Program

This supplemental material has been provided by the authors to give readers additional information about their work.

© 2025 Gonzalez CJ et al. *JAMA Network Open*.

eAppendix. Interview Guide: Engaging Hispanic Men in the Diabetes Prevention Program

Interview Guide (Applicable to those with low engagement)

Thank you for agreeing to discuss your thoughts on the diabetes prevention program. This interview is expected to take about 30 minutes of your time. There are no right or wrong answers to these questions, we are only interested in finding out your thoughts and feelings.

1) Tell me about when you first were informed that you had prediabetes, or that your blood sugar result was abnormal?
   a. What did your doctor tell you about pre-diabetes?
   b. What did your doctor tell you about how to treat pre-diabetes or lower your risk of diabetes?
   c. Besides speaking with or getting advice from your doctor, what else do you feel would help you lower your risk of developing diabetes?

2) Have you tried to lower your risk of developing diabetes before? What was that like?
   a. What things did you try doing?
   b. What things made it hard to make those changes?
   c. What things kept you motivated to maintain those changes?

3) What were you told about the diabetes prevention program? (**Jump to 4 if NA**).
   a. What would the program require from you?
   b. How would the program be helpful to you?

4) What do you think you should have been told about the diabetes prevention program?
   a Why do you believe that would help you decide in participating?
   b Who should have told you about the diabetes prevention program? Why?

5) What made you decide not to take part in a diabetes prevention program? (**Jump to** 6 if NA)
   a. What were your initial impressions when you heard about the program?
   b. Was there anything you are or were concerned about regarding the program?
   c. Is there anything specific that made you want to participate in the program?
   d. Did you discuss your diagnosis or decision with others? With whom?

6) From what you remember why would you not want to take part in a diabetes prevention program?
   a Was there anything you may have been concerned about regarding the program?
   b What challenges came or comes to mind when thinking about participating.
   c Did you speak to someone else before deciding to participate? Can you explain why?

7) What, if anything, would make you want to participate in a diabetes prevention program?
   a. Is there anyone that could have encouraged you to think more about participating in the program?
   b. If you could design your own program to reduce your risk of diabetes, what would it look like?
   c. If you had taken part in the program, what would you want an ideal session to look like?
   d. Is there someone else you would want to participate in the program with?

8) I'd also like to hear about what you, as a Hispanic man, would want from a program.
   a. What topics about preventing diabetes would you want to hear about or would be especially useful to you?
   b. How would you feel about being in program with only other Hispanic men?

9) What information would be helpful to have to better manage your pre-diabetes?

© 2025 Gonzalez CJ et al. *JAMA Network Open*.

<u>Interview Guide</u> (Applicable to those with high engagement)

Thank you for agreeing to discuss your thoughts on the diabetes prevention program. This interview is expected to take about 30 minutes of your time. There are no right or wrong answers to these questions, we are only interested in finding out your thoughts and feelings.

1) What made you decide to take part in a diabetes prevention program?
    a) What were your thoughts or ideas about pre-diabetes before going into the program?
    b) Was there anything you were concerned about?
    c) Had you previously tried to reduce your risk of diabetes before taking part in the program? What was that like?
    d) What were your thoughts and feelings when you heard you might participate in a program to educate you and support you in preventing diabetes?

2) Let's talk about your experiences in the program.
    a) Try to remember a typical session you attended during the program. Can you describe to me what that was like?
    b) How did the experience differ from what you were expecting, if at all?
    c) How was participating in this program different from speaking with or getting advice from your doctor?
    d) Over the course of your participation in the program, did anything change in how you thought about how you could prevent diabetes?

3) Often, people do not attend many sessions. So, now I want to get your understandings and thoughts about that.
    a) What things kept you motivated to stay involved in the program?
    b) What things challenged your involvement?
    c) Can you tell me why some people would drop out of the program?

4) I'd also like to hear about what your impressions of the program.
    a) Was there some part of the diabetes prevention program that was especially useful to you? Can you tell me about that?
    b) Being a Hispanic man, do you think the program recommendations applied to you?
    c) Was there some part of the diabetes prevention study experience that was difficult or uncomfortable for you? Can you tell me about that?
    d) What would you tell a friend if they asked about wanting to reduce their risk of developing diabetes?

5) Now I would like to know more about your social experiences during the diabetes prevention program.
    a) What was your relationship with others in the program like?
    b) Tell me more about the interactions between members in your group.
    c) Did you learn anything from being in the group that you might not have learned from an individual session?

[Interviewer: Please choose appropriate questions for the respondent based on whether they were in a male only or mixed-gendered DPP]

6) How did you feel about being in a group with [only men OR both men and women]?
    a) What were some strengths of the program including [only men OR both men and women]?
    b) What were some of the draw backs of the program including [only men OR both men and women]?
    c) Are there any topics that you feel came up specifically because the program included [only men OR both men and women]?
    d) Is there anyone else you would've liked to incorporate in the program?

© 2025 Gonzalez CJ et al. *JAMA Network Open*.

7) Think about your whole experience during this study from the referral process to today. Is there anything else you would like to tell us about how it went?
    a) Do you have any feedback you would have given to the person who led your diabetes prevention program?

General Follow Up Demographic Questionnaire

Before we complete this interview, there are some final questions I have:

1) What is your gender?
    a) Male
    b) Female
    c) Other

2) How old are you? ___

3) What is your race:

a. White	d. American Indian/Alaska Native
b. Black c. Asian	e. Hawaiian or Pacific Islander
c. Asian	f. Other

4) What country, or countries, are you and your family from?

a. Mexico	f. Belize	k. Colombia	p. Paraguay
b. Puerto Rico	g. El Salvador	l. Venezuela	q. Uruguay
c. Guatemala	h. Nicaragua	m. Peru	r. Bolivia
d. Cuba	i. Costa Rica	n. Chile	
e. Dominican Republic	j. Panama	o. Argentina	

5) How well do you think you speak English?
    a) Very well
    b) Well
    c) Not well
    d) Not at all

6) Were your born in the United States?
    a) Yes
    b) No

7) What is the highest school grade you completed?
    a) Did not complete High School
    b) Completed High School
    c) Some College
    d) Completed College

8) Do you have any of the following health conditions?

a. Diabetes	d. Any history of cancer	g. Kidney
b. High blood pressure	e. Heart problems	h. Lung Problems
c. High cholesterol	f. Liver problems	i. Problems with the immune system

9) Do you smoke?
    a) Yes

© 2025 Gonzalez CJ et al. *JAMA Network Open*.

b) No

10) How many people live in your home? (i.e. how many people live in the home you live in?)

a. Just me (1 total)
b. One other person (2 total)
c. 3 total
d. 4 total
e. 5 total
f. More than 5 people

11) How confident are you filling out forms by yourself?

a) Extremely
b) Quite a bit
c) Somewhat
d) A little bit
e) Not at all

Thank you very much for taking the time to speak with me; your experiences are very informative in better advising Hispanic men about managing their pre-diabetes. Thank you.

© 2025 Gonzalez CJ et al. *JAMA Network Open*.

eTable. Codebook: Engaging Hispanic Men in the Diabetes Prevention Program

Themes and Subtheme	Participant Engagement	Belief/ Contrasting Belief	Code
**C[a] Knowledge**	High Engagers	Moderation and balance are healthy	Things I learned from the DPP\Changing eating behaviors is about moderation and control
	Low Engagers	I have limited awareness of prediabetes and the DPP	Learned or Acquired Knowledge\Nutrition Guidance from my Health Team
			Pre-Existing\Clueless about next steps after prediabetes diagnosis
			Pre-Existing\Not exercising is harmful for my health
			Pre-Existing\The DPP made me aware of my prediabetes
		Healthy diets are restrictive diets	Pre-Existing\There's a certain diet to be healthy
			Pre-Existing\Unhealthy eating harms my health
**C[a] Mental Capacity & Behavioral Regulation**	High Engagers	Consistently choosing healthy options can be a cognitive load	Controlling food cravings is hard
			There is a difference in eating what you want versus what you need
	Low Engagers	I forget to eat well	Forget to eat healthy due to no symptoms
		Behavioral Regulation	I do frequent health diagnostic tests
			I exercise in manageable ways to be healthier
			I stopped drinking and smoking to better my health
**C[a] Skills**	High Engagers	Specific skills allow me to eat healthily and exercise	I'm familiar with exercising
			New skills I obtained from DPP\DPP taught me how to cook and eat well
			New skills I obtained from DPP\I can exercise on my own now
			New skills I obtained from DPP\Learned to read labels and count calories
	Low Engagers	I can think independently and have useful interpersonal skills	I can draw conclusions from new information
			I can help others with my life experience
			New skills I obtained from DPP\I gained communication skills from the DPP
**M[b] Beliefs about Capabilities**	Low Engagers	I am self-sufficient	I am capable of handling my health issues by myself
**M[b] Beliefs about Consequences**	Low Engagers	The risks of the DPP outweigh its benefits	The DPP will expose me to more risks than benefits

© 2025 Gonzalez CJ et al. *JAMA Network Open*.

Themes and Subtheme	Participant Engagement	Belief/ Contrasting Belief	Code
**M[b] Emotions**	High Engagers	Fear of diabetes	Fear and Relief Experienced\I fear the severe impacts of Diabetes
			Fear and Relief Experienced\I felt relief from joining the program
	Low Engagers	I am skeptical of my diagnosis and of doctors	Having diabetes is out of my hands
			Skepticism, Doctors always find something
		Emotions	I didn't feel well after hearing my diagnosis
			I feel relief from being supported and cared for
			Proper diet and exercise makes me feel better
**M[b] Goals and Intentions**	High Engagers	I want to avoid diabetes	I don’t want to be on medication
			I want to prevent the severe impacts of diabetes
	Low Engagers	I can think independently and have useful interpersonal skills	I want to be in a program to help others
		I want to better navigate healthcare	I want to learn how to navigate the healthcare system
**M[b] Reinforcement**	Low Engagers	DPP coaches teach what doctors do not	A guide can teach me what my doctor can’t
			I would be motivated to stay in a program with a guide
		Suggestions	Having these resources would make me want to participate more\Access to online resources would be helpful
			Having these resources would make me want to participate more\Additional health navigation would help
			Having these resources would make me want to participate more\Have financial incentives to reach goals
			Prefer over the phone outreach to participate
			Thoughts on what a program can do\Group oriented changes motivates me
			Structural changes I would like from the DPP

© 2025 Gonzalez CJ et al. *JAMA Network Open*.

Themes and Subtheme	Participant Engagement	Belief/ Contrasting Belief	Code
M[b] Social or Professional role and identity	High Engagers	The DPP should consider my Hispanic identity	DPP Considered Cultural Diet
			The Hispanic community's risk for diabetes is shocking
		The DPP should consider my male identity	I'd prefer a male only group\Its difficult to be vulnerable around women
			I'd prefer a male only group\men feel more comfortable to share among other men
			I'd prefer a male only group\Men wanted to go to the gym together
			Some comments were old school and misogynistic
			We're open to mixed groups for these reasons\Connection is more important than male vs mixed
			We're open to mixed groups for these reasons\The mixed-gender group was like any other group
	Low Engagers	The DPP should consider my Hispanic identity	A Hispanic only group would make me feel more comfortable
			As a Hispanic man it's my responsibility to deal with my health
			Hispanic people prefer small bits of info at a time
			I want to learn about diseases we are predisposed to
			I'm fine with an ethnically and racially diverse DPP
			In my Hispanic household we cook healthy
			The eating schedule my nationality is used to is different in the U.S
			There's an apprehensiveness to go to the hospital
O[c] Environmental context and resources	High Engagers	Finances impact my exercise options	Accessible food around me is unhealthy
			Accessing resources to exercise are expensive
		Accessing the DPP is challenging but feasible	It's difficult to get a hold of the DPP
			Virtual format for the DPP was challenging
			Work Inhibited my DPP Experience
	Low Engagers	DPP is inaccessible	Couldn't answer DPP call due to work
			DPP recruitment didn't call me back or inform me much
			Going somewhere far for the DPP would be challenging
			I don't have access to a computer
			Language barriers make navigating healthcare hard
			Remote learning would make accessing easier
		Finances impact my access to healthcare	Finances impact my access to healthcare

© 2025 Gonzalez CJ et al. *JAMA Network Open*.

Themes and Subtheme	Participant Engagement	Belief/ Contrasting Belief	Code
**O[c] Social Influences**	High Engagers	I act when my physicians tell me to act	Advice from your doctor is not received as well as from a friend
		Suggestions	Having People I know in the DPP would make me feel less lonely
		My friends/family have diabetes- I have to prevent it	I know people with diabetes so it's not shocking
			My family likes to get involved in my health
	Low Engagers	I act when my physicians tell me to act	It's encouraging when someone holds you accountable
			A doctor is the person who designates what to do
			My doctor wasn't alarmed so neither was I
		I want to avoid diabetes	I want to prevent diabetes for the sake of my family
		Prediabetes is socially normalized	Lack of Social Reinforcement Makes It Difficult to Make Change\It's difficult to change my behaviors when my social circle doesn't
			Lack of Social Reinforcement Makes It Difficult to Make Change\People tell me to be cautious of medical advice
			Lack of Social Reinforcement Makes It Difficult to Make Change\Prediabetes is normalized by people around me

[a]Rows labeled as "C" represent the COM-B domain Capabilities. [b]Rows labeled as "M" represent the COM-B domain Motivation. [c]Rows labeled as "O" represent the COM-B domain Opportunities.

© 2025 Gonzalez CJ et al. *JAMA Network Open*.
