# Supplementary material for: Engagement in the National Diabetes Prevention Program Among Hispanic Men
Source: JAMA Netw Open. 2025 Jun 18;8(6):e2515046. doi: 10.1001/jamanetworkopen.2025.15046 (PMC12177656; doi:10.1001/jamanetworkopen.2025.15046)
Supplement: Supplement 2. — Data Sharing Statement [file jamanetwopen-e2515046-s002.pdf]

Data Sharing Statement

Gonzalez. Engagement in the Diabetes Prevention Program Among Hispanic Men. *JAMA Netw Open*. Published June 18, 2025. doi:10.1001/jamanetworkopen.2025.15046

Data
Data available: Yes
Data types: Deidentified participant data
How to access data: cjg7003@med.cornell.edu
When available: With publication

Supporting Documents
Document types: Other (please specify)
Additional Information: Supplementary Materials
How to access documents: cjg7003@med.cornell.edu
When available: With publication

Additional Information
Who can access the data: Anyone can ask for the data.
Types of analyses: Any purpose.
Mechanisms of data availability: After approval of a proposal and signed data access agreement.
